# Individual and organizational interventions to promote staff health and well-being in residential long-term care: a systematic review of randomized controlled trials over the past 20 years

**DOI:** 10.1186/s12912-024-01855-7

**Published:** 2024-03-22

**Authors:** Michael Herz, Sabina Bösl, Doris Gebhard

**Affiliations:** 1 Department Health and Sport Sciences, TUM School of Medicine and Health, Technical University of Munich, Georg-Brauchle-Ring 62, 80992 Munich, Germany; 2 European Foundation for the Care of Newborn Infants, Hofmannstrasse 7A, 81379 Munich, Germany

**Keywords:** Health promotion, Nurse, Residential long-term care, Nursing homes, Systematic review

## Abstract

**Background:**

Staff in residential long-term care (RLTC) experience significant physical and mental work demands. However, research on specific interventions to promote staff health and well-being in RLTC facilities is limited. This systematic review aimed to synthesize the current evidence on health promotion interventions among RLTC staff.

**Methods:**

A comprehensive systematic literature review was conducted on studies published between January 2000 and April 2023. Four electronic databases were searched, including PubMed, Web of Science, Cochrane Central Register of Controlled Trials (CENTRAL), and PsychArticles via EBSCO. The review followed the guidelines outlined in the Preferred Reporting Items for Systematic Reviews and Meta-Analyses (PRISMA) protocol. The methodological quality of the included studies was assessed using the Risk of Bias Assessment tool (RoB 2).

**Results:**

A total of 26 publications, referring to 23 different interventions with a randomized controlled design were included. Among these interventions, ten used training/educational approaches, six used behavioral approaches, and seven employed a multimodal approach. Significant improvements in health and well-being outcomes were found in four interventions using a training/educational approach, three interventions using a behavioral approach, and four interventions using a multimodal approach. Within the interventions studied, twelve specifically targeted the reduction of job demands, while only one intervention exclusively addressed job resources among RLTC staff. Furthermore, ten interventions addressed primary outcomes that encompassed both job demands and job resources.

**Conclusion:**

Current evidence for health promotion interventions among RLTC staff is still limited, but research suggests that there is potential to improve certain outcomes related to RLTC staff health and well-being. Future research is recommended to contemplate a tailored intervention design that encompasses both individual-level and organizational-level approaches, and gender-specific physiological and sociological characteristics of RLTC staff. Moreover, detailed reporting of the development process, and research on the interaction between job demands and resources of RLTC staff are also recommended.

**Supplementary Information:**

The online version contains supplementary material available at 10.1186/s12912-024-01855-7.

## Background

Society in Western countries is aging [1, 2]. The EU projects that the number of people aged 65 years and older will increase from 92.1 million in 2020 to 130.2 million in 2050 [2]. A similar trend has been predicted for people aged 80 and over [1]. An aging society and increasing life expectancy are demographic factors that significantly affect long-term care (LTC) systems [3]. In the EU, the number of people potentially in need of LTC is projected to increase from 30.8 million in 2019 to 38.1 million in 2050 [3, 4]. LTC describes the support of people with significantly limited abilities in activities of daily living and is provided informally, formally, or through a mixture of both [5]. Formal LTC services are provided by trained nursing staff in residential or non-residential settings, including home care services, community (day) care services, and residential long-term care (RLTC) [4]. In the EU, 71.0% of the formal LTC workforce is employed in RLTC, while 29.0% work in non-residential care [4].

The formal LTC sector is currently facing significant challenges in recruiting new staff, as the nursing profession appears unattractive due to poor working conditions [3]. In addition, the workforce in LTC is aging, with most employees being between 50 and 60 years old (28.9%) [6]. The current age profile of the LTC workforce and the poor working conditions in formal LTC are contributing factors that could exacerbate the present staff shortage in the upcoming years [3].

Employees across the different LTC sectors have similar working tasks. However, recent research demonstrated that work-related health burdens for the employee depend on the specific characteristics of the care environment [7–9]. The nature of staff work in RLTC demonstrates a unique work environment within the formal LTC setting. In contrast to other care sectors, employees in RLTC must deal with residents that have a higher degree of care (e.g., assistance in activities of daily living), a higher degree of mobility impairments (e.g., bedriddenness, immobility), and a higher level of cognitive impairments (e.g., residents with dementia) [10–12]. These characteristics in RLTC are associated with a physically and psychosocially demanding working environment [3, 4, 13].

Physical working demands in RLTC are related to direct care activities and work organization. Nursing activities include working in forced positions, heavy lifting and carrying, and bathing overweight or bedridden residents [8, 14]. These working tasks occur frequently and repeatedly in their daily work routines, which are associated with an increased risk of musculoskeletal disorders [15]. A study by Cheung et al. [16] found that 88.4% of caregivers are affected by work-related musculoskeletal disorders. Further studies showed that musculoskeletal disorders can be associated with RLTC staff absenteeism, limited quality of life, and high costs for the company and the healthcare system [17, 18].

Further characteristics of work organization in RLTC can have a significant impact on the psychosocial demands which, in turn, can have a negative impact on their health and well-being [3]. Work in RLTC is organized in alternating shifts, including day, evening, and night shifts. These shifts include atypical working hours and irregular work rhythms, which can negatively impact the balance between personal (family) and work life, mental well-being, and quality of life [4, 19–23]. Night shifts, which is specific to the stationary care setting, can disrupt regular circadian rhythms, impacting the sleep-wake cycle [24]. RLTC staff experience significant pressure to meet performance expectations, handle disruptions, manage multiple tasks simultaneously, and maintain documentation requirements [25, 26]. These conditions contribute to a high workload and time pressure on a daily basis [4]. The increased time spent on documentation activities reduces the time available for direct resident care and other essential work tasks [27]. This lack of time and limited opportunities for recovery during the workday are associated with psychological distress and the development of burnout among RLTC staff [28], which was underscored by a cross-sectional study indicating a 40.0% prevalence of caregiver burnout [29].

Working in RLTC encompasses complex interpersonal relationships with residents that can last for several years, requiring 24-hour constant and intensive care. Staff is challenged to establish clear boundaries with residents, which can be difficult given the nature of the RLTC setting. Within this bond, staff must deal with demanding psychosocial issues related to residents’ behavior, end-of-life, and suffering [30]. Additionally, RLTC staff may encounter aggressive behavior from residents, which can contribute to experiences of different kinds of violence in the workplace [31, 32].

Besides the physical and psychosocial demands, the literature indicates that job-related resources can positively impact the health and well-being of RLTC staff [33]. Research on health-promoting resources in RLTC staff is still in its infancy. However, some studies have already demonstrated that higher levels of supervisor support [34], social support [35], and leadership [36] are health-promoting factors that contribute to better health and well-being among RLTC staff.

Both job-related demands and resources shape the daily work of staff in RLTC and influence their health and well-being. Their impact on health and the interaction between demands and resources has been substantiated by applying theoretical models across various nursing professions [37, 38]. One of these models is the job demands-resources model (JD-R model), which posits that the health and well-being of employees are contingent upon the balance between the demands and resources experienced within their work environment [33, 39]. According to this model, job demands are physical, psychosocial, or organizational factors that arise due to the workplace’s specific characteristics and negatively impact the individual. Job resources exert a dual impact on health and well-being: firstly, by directly impacting workers’ motivation and work engagement, and secondly by acting as a protective buffer against the adverse effects of job demands. It is important to note that when the perceived job demands outweigh the available job resources, it can lead to undesirable outcomes such as early retirement, presenteeism (being present at work but not fully productive), and turnover among RLTC staff [40–42].

Thus, the JD-R model emphasizes that measures to promote health and well-being and mitigate early retirement must consider both the reduction of job demands and the enhancement of job resources. Keeping RLTC staff in their job for as long as possible is critical to reduce staffing shortages as they commonly believe that they are unable to continue fulfilling their job responsibilities beyond the age of 60 [4]. Several OECD countries have introduced strategies to attract and retain staff in the RLTC sector. In Germany, health insurance funds are required by law to provide financial support for the implementation of workplace health promotion programs in RLTC [43]. Workplace health promotion programs aim to improve employees’ health and well-being by changing the workplace’s structure and environment, encouraging active participation, and supporting personal development [44]. Several studies have shown workplace health promotion interventions’ effectiveness (e.g., improving mental and physical health) in various occupational settings [45–47]. Workplace health promotion interventions in nursing have become an important approach to improving health and well-being in recent years [48–50]. Existing systematic reviews of workplace health promotion in nursing focus on specific intervention types, such as stress-management [51], physical activity [52], mindfulness-based stress reduction [53], and physical exercise training [54]. However, most systematic reviews in nursing are often targeted at hospital staff or include nursing staff without differentiating the care setting. Recently, Schaller et al. [55] presented a review of existing workplace health promotion interventions in various nursing settings (e.g., acute medical care hospitals, nursing homes, and home-based LTC) in Germany. Moreover, a systematic review by Gebhard & Herz [56] examined interventions aimed at improving the health of home care workers.

Based on the given literature, it is recommended that the health and well-being of staff in RLTC need to be addressed through health promotion interventions tailored to their specific needs [55, 57, 58]. However, there is currently a lack of knowledge regarding the availability of interventions that specifically target the health and well-being of RLTC staff. To date, no systematic review has been conducted with a focus on health promotion interventions for this specific population. The purpose of this systematic review is to present the current evidence on the impact of health promotion interventions [44] on health and well-being among RLTC staff. Therefore, the objectives of this systematic review were to:

1. identify the intervention approaches that have been used to address the health and well-being of RLTC staff,
2. identify the effectiveness of health promotion interventions on the health and well-being of RLTC staff,
3. determine which demands and resources have been addressed as primary outcomes,
4. derive recommendations for the development of tailored health promotion interventions for RLTC staff.

## Methods

This systematic review was conducted in accordance with the Preferred Reporting Items for Systematic Reviews and Meta-Analyses (PRISMA) guidelines [59]. This study was registered with the PROSPERO database (registration number: CRD42020203911).

### Literature search

Two consecutive computerized systematic searches were conducted in the electronic databases: PubMed, Web of Science, Cochrane Central Register of Controlled Trials (CENTRAL), and APA PsycArticles. The initial literature search was conducted on 15 July 2020 and was restricted to publication period (year: 2000–2020), language: English or German, and articles published in peer-reviewed journals. An updated literature search was conducted in the abovementioned databases on 12 April 2023. This search was additionally restricted to randomized controlled trials, because the initial literature search resulted in an acceptable number of randomized controlled trials. Therefore, we decided to include only RCTs in this systematic review. Keywords were collected through expert opinion, literature review, and controlled vocabulary (e.g., Medical Subject Headings [MeSH]). The Boolean search syntax for each respective database is listed in Supplementary Material 1. The reference lists of articles were searched for potentially relevant articles. The search results from all databases were exported and transferred into the software Rayyan [60]. Duplicates were identified and removed using Rayyan and Endnote software. The initial search results were independently screened by two researchers (SB and DG), and the consecutive literature search was independently screened by two researchers (SB and MH). Any disagreements were resolved by consensus-based discussion between the involved researchers. Potentially relevant articles were initially screened based on their titles and abstracts, following the eligibility criteria, and subsequently, full-texts were examined. In case of any disagreement regarding study eligibility, a third researcher (MH or DG) was consulted for clarification, or a consensus-based discussion was held.

### Eligibility criteria

A PICOS (population, intervention, comparator, outcome, and study design) approach was used to identify relevant publications for inclusion. Given the aim of this systematic review to provide current evidence of health promotion interventions to improve the health and well-being of RLTC staff, we decided to include publications according to the following criteria: (1) studies conducted with staff in RLTC, (2) behavioral, training/educational, or organizational interventions or program whose major component is aimed at improving personal/occupational health and well-being of RLTC staff, (3) studies with a control group including active/passive control condition, waitlist, or usual practice, (4) studies with primary outcomes aiming at personal/occupational health and well-being of RLTC staff, and (5) studies with any randomized controlled design (e.g., parallel, cluster, stepped-wedge cluster). Conversely, review articles, protocol papers, book chapters, letters to the editor, and non-peer-reviewed articles were excluded.

### Data extraction

Two independent reviewers (MH and SB) extracted data from the included studies with a standardized predefined Microsoft Excel sheet. In case of any disagreement regarding data extraction, DG was involved for clarification. The following information was retrieved, if possible, from every eligible study: (1) study identification: first author, publication year, country (2) study design (control condition), (3) participants characteristics (baseline): job title, sample size in each group, mean age, gender distribution, (4) intervention modalities: intervention description, intervention duration, (5) measures, (6) primary outcomes related to personal/occupational health and well-being, and (7) results. To delineate the treatment effect, we systematically retrieved the reported effect sizes, including Cohen’s d and Eta-squared (η2), where available.

### Classification of intervention approaches

We decided to categorize the intervention types of the included studies in terms of their intervention approach. Drawing from the work of Gebhard & Herz [56], we categorized interventions into three distinct approaches: (1) behavioral interventions, (2) training/educational interventions, and (3) organizational interventions. Both the behavioral and training/educational approach primarily focuses on the individual level, while the organizational approach emphasizes interventions at the organizational level. According to Westermann et al. [61], we added a further intervention approach: (4) the multimodal intervention approach, which includes measures that incorporate at least two of the above-mentioned intervention approaches.

Behavioral interventions are targeted to change the behavior of an individual to promote and encourage health-related behavior. This approach includes the education of individual skills or techniques to cope with the negative impact of stressors on the health status during and outside the workplace [62]. For instance, these interventions might involve measures promoting physical activity, a healthy diet, or stress-management techniques.

Training/educational interventions aim to increase employees’ abilities to cope with specific stressors related to their work tasks, working environment, or working conditions. These interventions focus on changing personal (individual behavioral) characteristics (e.g., work-related competencies) to improve employees’ functioning at the workplace [63].

Organizational interventions aim to improve working conditions and create a healthier work environment. These interventions primarily focus on making factual changes to work structures, working conditions, reducing the intensity of workloads, increasing employees’ participation in decision-making, and improving teamwork. By addressing the underlying factors contributing to stress and strain in the work situation, organizational interventions strive to create a supportive and conducive environment for employee health and overall well-being [62].

### Risk of bias assessment

Three researchers (MH, SB, and DG) independently conducted the methodological quality assessment of the included studies using the revised version of the Cochrane risk of bias (RoB 2) tool for RCTs and cluster RCTs [64]. Any disagreements with quality rating were resolved by consensus-based discussion. The risk of bias was assessed across five domains: (1) Risk of bias arising from the randomization process, (2) Risk of bias due to deviations from the intended interventions (effect of assignment to intervention), (3) Risk of bias due to missing outcome data, (4) Risk of bias in the measurement of the outcome, and (5) Risk of bias in the selection of the reported result. For cluster RCTs, a further domain was assessed concerning the risk of bias arising from the timing of identification or recruitment of participants. Each domain can be rated as “low”, “moderate”, or “high” risk of bias. An overall risk of bias judgment was conducted across all domains for each included study. The robvis tool was used to visualize the risk-of-bias plots in parallel and cluster RTCs [65].

## Results

**Fig. 1:**
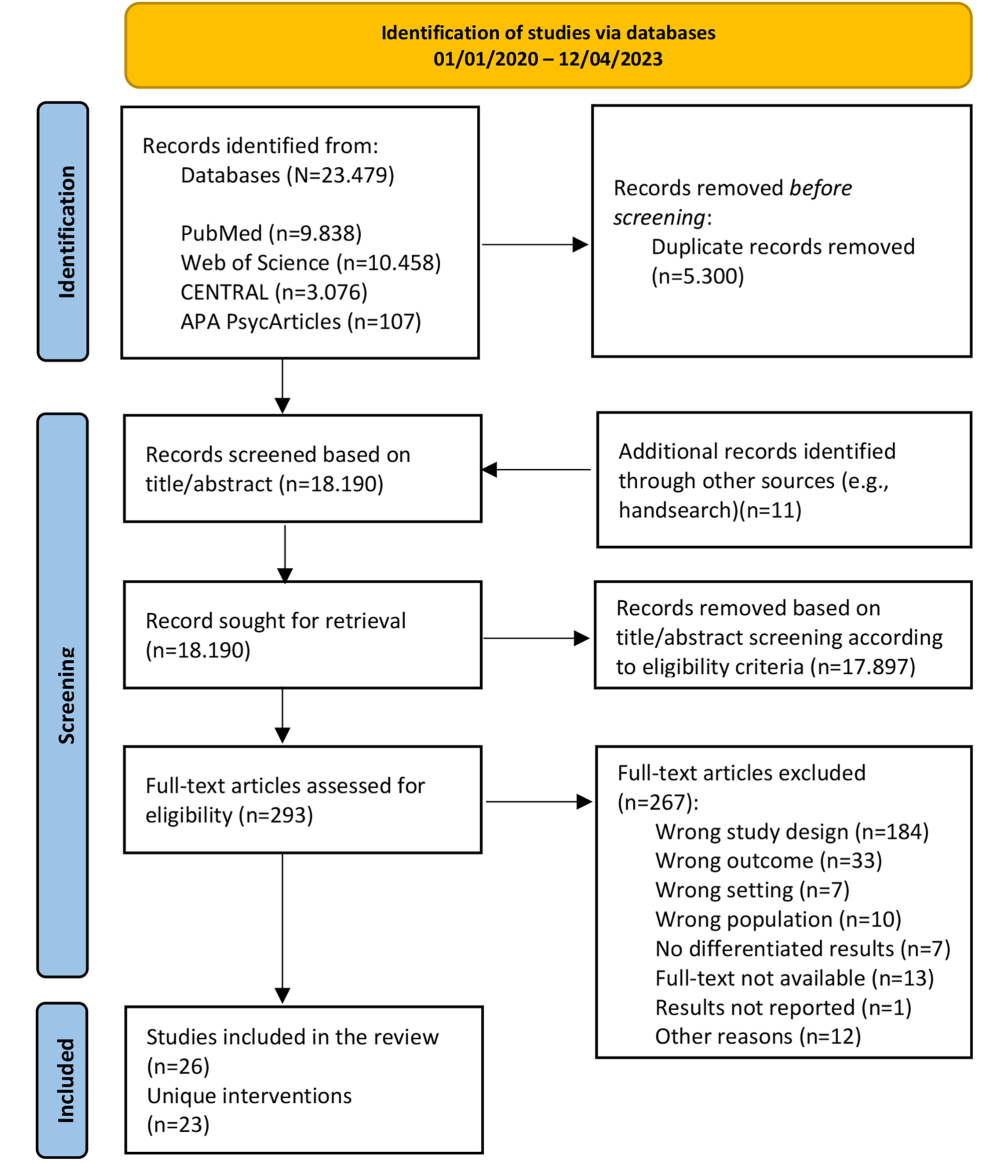
PRISMA flow diagram of this systematic review

In total, the literature search yielded 23,479 articles by the searches. All titles and abstracts were screened and 293 full-texts were assessed for inclusion, resulting in *n* = 26 included publications and *n* = 23 different interventions for the narrative literature synthesis. The study selection process is provided in Fig. 1.

### Study characteristics

Characteristics of the included studies are presented in Table 1. Eligible studies were published between 2003 and 2022. Health promotion interventions for staff in RLTC were found in high-income and upper-middle-income countries. The majority of studies was conducted in the United States of America (*n* = 8) [66–75]. Four interventions were conducted in Australia [76–79], three in the Netherlands [80–82], two in Norway [83, 84], and one each in Denmark [85], Italy [86], Japan [87], Mexico [88], Spain [89], and Portugal [90, 91].

**Table 1:** Characteristics of included randomized controlled trials

Author, year, country	Study Design (control)	Participants	n; mean age (SD)^a^	Gender (female %)^a^	Intervention description	Duration	Measure (s)	Primary outcome (s)	Results (Post [ES] / (Follow-up [ES])^b^
**Intervention type: Behavioral approach (** ***n*** ** = 6)**									
Balk-Møller et al. (2017), Denmark	RCT (usual practice)	RLTC staff	IG (152); 47.0 (10.0) CG (117); 47.0 (9.9)	IG: 92.1 CG: 92.3	**SoSu-life: A Web- and app-based tool**: -Suggestions for activities and programs, practical tips and tricks -Social features: weekly assignments, colleague challenges -Participants chose pledge at the beginning: lose weight, eat healthier, improve physical fitness, improve physical strength, quit smoking, decrease no of cigarettes smoked, maintain healthy lifestyle -Subsequent communication—feedback, frequency, and content of emails and text messages provided by the program, and topics for user-to-user communication, Messages contained information about specific health issues related to the pledge, general tips, and tricks on health and well-being -Initial 16-weeks included team competition, subsequent 22 weeks without team competition	16 to 38 weeks	Digital electronic scale	Body weight	+[NA]/+[NA]
Brox & Foystein (2005), Norway	RCT (usual practice)	Nurses and nurse aides	IG (63); 42.5 (NA) CG (64); 42.5 (NA)	IG: 97.0 CG: 96.0	**Physical exercise program**: -Light aerobic exercise classes -Classes regarding nutrition and stress management	24 weeks	UKK walking test COOP/WONCA Community insurance register	Cardiorespiratory fitness Overall Health Sickness absences	- - -
Kamioka et al. (2010), Japan	CRT (usual practice)	Nurses	IG (44); NA CG (44); NA	IG: 100 CG: 100	**Education and exercise program**: -Lecture contained information on risk factors, biomechanics of care-movement, treatment, and recommended exercise, eight elements of stretching, leaflet for monitoring -DVD of the series of exercises was distributed to each nursing home	12 weeks	10-cm Visual Analogue Scale	Pain	-
Kloos et al. (2019), Netherlands	CRT (usual practice)	Nursing staff	IG (79); 39.6 (13.0) CG (49); 44.7 (10.0)	IG: NA CG: NA	**Online multicomponent Positive Psychology intervention**: -8 modules, key topics: positive emotions, discovering and using strength, optimism, self-compassion, resilience, positive relations -> each module consists of psychoeducation and five exercises -One lesson per week advised	8 weeks	Mental Health Continuum-Short Form Maastricht Job Satisfaction for Healthcare Utrecht Work Engagement Scale	Well-being Job satisfaction Work engagement	- +[0.10]^e^ -
Perez et al. (2022), Spain	CRT (wait-list)	Nurses	IG (35); NA CG (39); NA Total sample: 37.0 (9.13)	IG: NA CG: NA Total sample: 89.6	**Online Mindfulness-based intervention**: -Elements of mindfulness-based stress reduction and mindfulness-based cognitive therapy -Main characteristics: six recorded sessions of 60 min, videos, interactive exercises, relaxation and breathing techniques, individual reflective writing exercise -Assignments to practice in daily life -Weekly sessions, tasks, and audio-guided meditation were provided -Users were informed via notifications	6 weeks	Professional Quality of Life Scale Spanish Adaptation	Compassion satisfaction Burnout Compassion fatigue	- / - +[NA] / +[NA] +[NA] / +[NA]
Riello et al. (2021), Italy	RCT (alternative activity)	Medical/healthcare, administrative, technical personnel	IG (119); NA CG (119); NA	IG: 87.4 CG: 89.1	**Self-help plus (SH+) program**: -Teaching a series of psychological techniques to cope with stress or promoting reflection on relevant topics such as work roles and related emotions -Distribution of an individual self-help audio-visual tool -SH + includes a pre-recorded audio course, originally meant to be delivered by facilitators in a group setting and an illustrated self-help book	1 week	Impact of Event Scale-Revised Generalized Anxiety Disorder Scale	Subjective distress Anxiety symptoms	- / - - / -
**Intervention type: Training/educational approach (** ***n*** ** = 10)**									
Barbosa et al. (2015), Portugal	CRT (education only)	Nursing assistants	IG (27); 43.4 (10.0) CG (31); 45.9 (8.0)	IG: 100 CG: 100	**Person-Centered Care-Based (PCC) Psychoeducational intervention**: -Education component aimed to provide nursing assistants with: (1) principles to integrate person-centered care within the care routines (2) basic knowledge about dementia (3) PCC-based interaction strategies; in the three days after session, support of experts during morning care	8 weeks	Perceived Stress Scale 12 Maslach Burnout Inventory Minnesota Satisfaction Questionnaire	Perceived stress Burnout Job satisfaction	- - -
Barbosa et al. (2016) Portugal							Perceived Stress Scale 12 Maslach Burnout Inventory Minnesota Satisfaction Questionnaire	Perceived stress Burnout Job satisfaction	6-month follow-up - - -
Bieldermann et al. (2021), Netherlands	CRT (usual practice)	Nursing staff	IG (168); 41.1 (12.2) CG (124); 40.3 (10.4)	IG: 96.4 CG: 96.9	**The educating Nursing Staff Effectively (TENSE) program:** -3-day workshop (2 sessions of 2.5 h), followed by 2 follow-up sessions (2 sessions of 2.5 h) Content of the educational program: -Providing knowledge about dementia -Educating about the origins of challenging behavior and how to manage it -Educating how to recognize distress caused by challenging behavior in yourself and in colleagues -Practicing general behavior management skills that can be used in approaching the resident -Practicing how to signal signs of impending challenging behavior	24 weeks	Utrecht Burnout Scale - C	Burnout: Emotional exhaustion Depersonalization Personal accomplishment	- / - - / - - / -
Bramble et al. (2011), Australia	CRT (education only)	Nursing staff	IG (31); 39.8 (8.5) CG (27); 41.2 (8.4)	IG: 87.1 CG: 92.6	**Family involvement in care (FIC) - Education program**: -First session: information about dementia -Second session: problems faced by family members of residents with dementia in RLTC -Third session: how to negotiate with family caregivers to increase involvement in care; introduction of partnership contract agreement role negotiation, therapeutic communication, conflict resolution skills	4 weeks	Caregiver Stress Inventory Staff Perceptions of Caregiving Role Knowledge of Dementia Test Attitudes Towards Family Checklist	Experienced stress Experienced stress Knowledge of dementia Attitude towards family	NA / NA NA / NA NA / NA NA / NA
Chenoweth et al. (2014), Australia	CRT (usual practice)	Nursing staff	IG (17); NA CG (18); NA	IG: NA CG: NA	**Humor intervention**: -Staff attended trainings sessions with experienced humor performer the intervention compromised humor sessions among professional performers, nursing staff, and residents	12 weeks	Self-developed questionnaire	Well-being	- / -
Davison et al. (2007), Australia	CRT (usual practice)	Registered Nurses, unlicensed Nursing assistants	IG I (29); NA IG II (35); NA CG (26); NA Total sample: 45.0 (11.0)	IG I: NA IG II: NA CG: NA Total sample: 90.0	**IG I: Dementia training program + peer support** -Combination of didactic and experiential learning, -skills to use in caring for residents with dementia-related behaviors, peer support program was to facilitate informal group support, whereby staff members could discuss challenging behaviors, their subsequent emotional reactions and how to cope with work-related stress **IG II: Dementia training program only**	24 weeks	Maslach Burnout Inventory Self-Efficacy of Dementia Care	Burnout Self-efficacy	IG I: - / - IG II: - / - IG I: +[NA] / +[NA] IG II: - / -
Irvine et al. (2007), USA	RCT (usual practice)	Nurse Aides	IG (34); NA CG (28); NA	IG: 88.2 CG: 82.1	**Internet-based interactive multimedia training**: -Skills to approach an agitated resident showing potentially dangerous behaviors, skills to de-escalate situations -Intervention strategy: A.I.D. - approach: Assess, Investigate, Do Something -> emphasis on person-centered approach -> content was delivered online via video vignettes, video clips, voice over clips, and video testimonials	NA	Self-developed questionnaire Self-developed questionnaire Self-developed questionnaire Self-developed questionnaire Self-developed questionnaire	VST- knowledge VST self-efficacy Attitudes Self-efficacy Behavioral intentions	+ [0.24]^f^ + [0.33]^f^ + [0.40]^f^ + [0.36]^f^ + [0.17]^f^
Leontjevas et al. (2020), Netherlands	stepped-wedge CRT (usual practice)	RLTC staff and other disciplines	IG (NA); NA CG (NA); NA Total sample: *N* = 796; 39.4 (11.9)	IG: NA CG: NA Total sample: 96.0	**The Act in case of Depression program:** -3-hour training workshop -Evidence-based pathways for screening, identifying, diagnosing, treating, and monitoring depression -Consists of three components: structured assessment with a two-step screening and diagnostic procedure, three multidisciplinary treatment modules, and monitoring of treatment results	NA	8-item Scale by de Jonge (1995) Maastricht Work Satisfaction Scale for Healthcare Questionnaire on Experience and Assessment of Work	Job demands Job satisfaction Workplace autonomy	- - -
O’Brien et al. (2019), USA	RCT (wait-list)	Nurses and nurse aides	IG (37); NA CG (34); NA Total sample: 37.9 (13.2)	IG: NA CG: NA Total sample: 86.0	**Group-based Acceptance and Commitment intervention**: (1) acquisition and practice of acceptance, mindfulness, and cognitive delusion techniques (2) values identification and how they can be embedded in daily work tasks; (3) making explicit commitments to changing work behaviors that interfere with values	2 weeks	Risk for Nursing Assault and Injury Inventory General Health Questionnaire	**Work environment** Workdays loss Injury frequency Musculoskeletal complaints Mental health symptoms	+[0.01]^f^ - - +[0.08]^f^
Pillemer et al. (2003), USA	CRT (usual practice)	Nursing staff	IG (256); NA CG (399); NA	IG: 92.2 CG: 94.7	**Cooperative communication intervention:** -1 day workshop (7 h) (1) active/empathic listening skills. Focusing on identifying ‘‘communication helpers’’ that encourage others to express their opinions and feelings. (2) Feedback. Focusing on providing verbal cues to a conversation partner that allow a person to know how a message is received, permitting the speaker to adjust the message as needed. (3) I-messages. Focusing on using the first-person singular to express a problem or complaint.	8 weeks	Family Behaviors Scale Family Empathy Scale Maslach Burnout Inventory Self-developed question	Perception family behavior Perception family empathy Burnout Intention to quit	+[NA] / - NA - / - +[NA] / -
Torres-Castro et al. (2022), Mexico	CRT (usual practice)	RLTC staff, Health professional, Managers	IG (57); 36.4 (12.2) CG (39); 39.5 (12.6)	IG: 70.0 CG: 85.7	**Programmed for Optimizing Care in Dementia (PROCUIDA-Demencia) intervention:** -2-day training workshop: (1) Understanding challenging behavior, managing stress and distress, ways to improve communication and interaction (2) Team group work and organizational psychology elements to improve inter-personal skills (3) A set of psychosocial measures including doll therapy, psychomotor dance therapy, and reminiscence therapy	12 weeks	Maslach Burnout Inventory Approaches to Dementia Questionnaire Sense of Competence in Dementia Care Staff	Burnout Attitudes towards people with dementia and their care Level of staff sense of competence	- - -
**Intervention type: Multimodal approach (** ***n*** ** = 7)**									
Doran et al. (2018) I, USA	CRT (education only)	Nurse aides, nurses, housekeeper dietary, service workers, concierge staff, activities staff, kitchen staff, maintenance	IG (48); NA CG (50); NA	IG: 84.8 CG: 93.9	**Worksite Heart Health Improvement Project (WHHIP)**^**c**^: -Component 1: Environment and policy assessment -> recommendations for workplace (environmental) changes -Component 2: Education -> 30 min group education session on cardiovascular disease and health -Component 3: Motivation and active engagement -> Individual goal-setting, health competitions, social support dyads, testing of healthy foods, education on healthy eating, group sessions discussing exercise, diet, stress management modifications, group sessions -Component 4: Technology enhanced motivation -> 96 motivational text messages over 8 months -Component 5: Booster and long-term adherence -> Weekly booster sessions on motivation and engagement to improve cardiovascular health; mentoring and fostering of dyad partners	36 weeks	Depression-Anxiety-Stress Scale Modified Block Dietary Fat Screener Modified Block Dietary Fat Screener Pittsburgh Sleep Quality Index	Mood Sodium intake Fat intake Sleep quality	- / +[NA] / +[NA] - / +[NA] / - - / - / - - / - / +[NA]
Doran et al. (2018) II, USA							Digital blood pressure manometer Digital blood pressure manometer Finger-prick blood samples BMI Formula Pedometer	SBP DBP Cholesterol BMI Physical activity (steps)	- / - - / + [NA] - / - - / + [NA] +[NA] / +[NA]
Flannery et al. (2012) I, USA	CRT (education only)	Nursing assistants	IG (24); 43.3 (13.1) CG (15); 39.4 (13.1)	IG: 100 CG: 100	**Worksite Heart Health Improvement Project (WHHIP)**^**c**^: -Component 1: Environmental and policy assessment Assessing organizational, community, and policy factors affecting nursing assistants’ health -Component 2: Education of nursing assistants Providing foundation for cardiovascular disease prevention, encourage nursing assistants to make exercise and diet changes at work -Component 3: Ongoing Motivation of nursing assistants Motivating nursing assistants to exercise and reduce dietary fat and salt intake, actively engaged nursing assistants in physical activity and healthy eating	12 weeks	Nursing Home Administration Job Satisfaction Questionnaire Effort-Reward-Imbalance questionnaire Work ability index	Job satisfaction Effort Reward Over commitment Work ability	- / - - / - - / - - / - +[NA] / +[NA]
Flannery et al. (2012) II, USA							Exercise Self-efficacy Self-efficacy for health-related Diet Pedometer Digital blood pressure manometer Digital blood pressure manometer Finger-prick blood samples BMI Formula The Center for Epidemiologic Studies Depression Scale	Exercise self-efficacy Self-efficacy for health- related diet Physical activity SBP DBP Cholesterol BMI Depressive symptoms	- / - - / - - / - +[NA] / +[NA] - / - +[NA] / +[NA] - / - +[NA] / +[NA]
Jeon et al. (2015), Australia	CRT (usual practice)	RLTC staff	IG (202); 46.5 (NA) CG (301); 47.1 (NA)	IG: NA CG: NA	**Clinical Leadership in Aged Care (CLiAC) program**^**d**^: -Structured education and support program, designed to promote safe, high-quality person-centered and evidence-based care by assisting middle managers to develop effective team relationships and person/client-centered leadership strategies that enable them to deal with the day-to-day realities of care service -Participants received a set of learning resources for team building activities -Developing team-based action plans, providing education sessions	48 weeks	Work Environment Scale - R Multi-factor Leadership Questionnaire Organization register	**Work environment** Involvement Peer cohesions Supervisor support Autonomy Task orientation Work pressure Clarity Control Innovation Physical comfort **Management Leadership support** Transformational Transactional Passive avoidant Leadership Turnover rate	- / - - / - - / +[NA] - / - - / - - / - - / - - / - - / - - / - - / +[NA] - / +[NA] - / +[NA] - / +[NA] - / -
Kossek et al. (2019), USA	CRT (usual practice)	Nursing staff, Managers	IG (420); NA CG (511); NA	IG: NA CG: NA Total sample: 92.3	**STAR (Support, Transform, Achieve, Results) intervention**^**d**^: -Leader family & work support training -Work site facilitator-led participatory ROWE (Results-oriented Work Environment) training -Supervisory training on strategies to demonstrate support for employees’ personal and family lives while also supporting employees’ job performance -Participatory training sessions to identify new work practices -Activities included employee group sessions, after-session work-improvement redesign, leader computer-based training, behavioral self-monitoring by leaders and coworkers	16 weeks	K-6 Mental Health Screening Questionnaire Perceived Stress Scale 4	Psychological distress Perceived stress	- / - - / -
Marino et al. (2016), USA	CRT (usual practice)	Nursing staff, Managers	Nursing home staff: IG (652); 37.8 (12.7) CG (568); 38.9 (12.3) Manager: IG (33); 43.2 (9.2) CG (32); 45.9 (11.9)	Nursing home staff: IG: 93.7 CG: 91.7 Manager: IG: 93.9 CG: 90.6	**STAR (Support, Transform, Achieve, Results) intervention**^**d**^: -4 sessions for all employees and managers together -> Increase of support for coworkers, more results-oriented work culture, eliminating negative judgement ->Collective self-monitoring of their experiences and how often they applied the learned techniques -3 sessions for managers and supervisors only -> Provision of an handheld device with an application -> possibility to focus on specific supportive behavior e.g. Creative Work-Life Management for two weeks plus feedback on goal progress	16 weeks	Wrist-worn sleep monitor	Nursing home staff: Mean total sleep time Nighttime sleep duration Wake after sleep onset Number of Naps Nap duration Manager: Mean total sleep time Nighttime sleep duration Wake after sleep onset Number of Naps Nap duration	- / - - / - - / - - / - - / - - / - - / - - / - - / - - / -
Pillemer et al. (2008), USA	CRT (usual practice)	Nursing assistants	IG (379); NA CG (383); NA	IG: 93.4 CG: 92.4	**Retention specialist program**^**d**^: -Staff member was selected to be facility retention specialist -> advocate & implement programs to improve staff retention and commitment throughout the facility -Component 1: specialized retention training -Component 2: ongoing technical assistance -Component 3: leveraging community resources	48 weeks	Self-developed questionnaire Self-developed questionnaire Generic Job Satisfaction Scale Self-developed question Organization registers Self-developed question	Attitudes toward the facility Perceived Facility Retention Effort Job satisfaction Job stress Turnover rate Intention to Quit	+[NA] / - - / +[NA] - / - - / - - / +[NA] - / +[NA]
Tveito et al. (2009), Norway	RCT (wait-list)	RLTC staff and other disciplines	IG (19); NA CG (21); NA	IG: 100 CG: 100	**Integrated Health program**^**c**^: -Physical exercise, health information/stress management training & practical examination of workplace -One hour three times a week, consisted of: boy awareness, warm-up/aerobics/ergonomics, cool-down exercises, strength/stabilizing, stretching, relaxation -15 h of information on stress, coping, health and lifestyle & practical examination of workplace -Health and lifestyle information focused on exercise, nutrition, sleep, smoking, activity and musculoskeletal problems. The stress management training focused on both the positive and negative consequences of stress and how to cope with stress.	36 weeks	Subjective Health Complaints Inventory Organization register	Subjective somatic and psychological complaints Sick absence	- / NA - / -

a– characteristics at baseline; b - The plus sign (+) indicates that the intervention group was statistically significantly superior to the control group over time. The minus (–) sign indicates that the null hypothesis was upheld; in case of significant group x time effects the effect size is indicated if possible; ES– effect size; c– multimodal approach: behavioral and organizational approach; d– multimodal approach: training/educational and organizational approach; e– Cohen’s d, f– Eta-squared, NA– not available; RCT– randomized controlled trial; CRT– cluster randomized controlled trial; RLTC– residential long-term care; IG– intervention group; CG– control group; SBP– systolic blood pressure; DBP– diastolic blood pressure; BMI– body mass index

### Study design

The majority of studies applied a cluster randomized controlled design (*n* = 16). A parallel randomized controlled study design was applied in six studies [70, 73, 83–86, 89], and one study used a stepped-wedge cluster randomized controlled trial [80].

### Study sample

RLTC staff with primarily care activities (e.g., nursing staff, nurse aides, nursing assistants) was most frequently targeted (*n* = 15) [68–71, 73–77, 81, 82, 84, 85, 87, 89–91], and six interventions included nursing staff and other disciplines (e.g., kitchen staff, service workers, activity staff, managers) within the RLTC setting [66, 67, 72, 78, 83, 86, 88]. One intervention targeted RLTC staff without any specification [79]. Sample sizes at baseline ranged from 35 [77] up to 1.258 [72] participants with an average sample size of 447 participants across the included studies. In total, this systematic review comprises a sample size of 6,795 participants across the included studies. The mean age of the study groups ranged from 36.4 to 47.1 years. In ten studies the mean age of the study groups was not appropriately reported [66, 67, 70, 71, 74, 75, 77, 83, 86, 87, 89]. Women were predominated in the sample (ranging from 70.0 to 100% of the total sample). Torres-Castro et al. [91] investigated the highest percentage of male participants with 24.0%. In three studies, the gender distribution of the sample was not reported [77, 79, 81].

### Risk of bias assessment

The overall risk of bias judgment of cluster randomized trials showed that most of the trials were rated as “some concerns” (see Supplementary Material 2). One study was considered with a low risk of bias [79] and three studies with a high risk of bias [69, 77, 78]. Most studies did not provide sufficient information in the domain of the randomization process and thus were considered as “some concerns”. Selection bias of the reported result was assessed in several studies with “some concerns” because no study protocol or pre-specified analysis plan was available. The overall risk of bias judgment of parallel randomized controlled trials showed that four trials were rated as “some concerns” and one study was rated as high risk of bias (see Supplementary Material 2). No study protocol or pre-specified analysis plan was available for one of the parallel randomized controlled trials.

### Characteristics of the intervention approaches

Our results revealed that ten out of 23 interventions used a training/educational approach, seven interventions used a multimodal approach, six interventions used a behavioral approach, and no study applied an organizational approach.

#### Training/educational approach

An educational training program was implemented in six interventions to address challenging resident behavior by enhancing work-related competencies, skills, and behavior. These interventions specifically targeted dementia-related [76, 78, 82, 88, 90, 91], and depression-related [80] resident behaviors. Additionally, one intervention focused on equipping RLTC staff with skills to de-escalate aggressive resident behavior situations [70]. Two interventions aimed to be implemented with residents to allow RLTC staff to build meaningful relationships with residents that can positively impact their health and well-being. In one intervention, a humor intervention was implemented to teach RLTC staff specific techniques and skills for incorporating humor and play into daily daycare activities [77]. In the second study RLTC staff was trained to conduct psychosocial programs such as doll therapy, dance-based psychomotor therapy, and reminiscence therapy with the residents [88]. Two studies focused on general techniques to deal with challenging tasks related to communication with relatives, coworker support, and stressful situations at the workplace [73, 75].

#### Behavioral approach

In two studies, the intervention focused primarily on promoting physical activity and exercise training (e.g., aerobic, strengthening, or stretching exercises) with additional health education [84, 87]. In one of those the intervention supplemented the exercise program with additional health information on diet and stress-management [84], while the other intervention provided information specifically on low back pain pathology [87]. One study implemented a lifestyle program using a web- and app-based tool that addresses various health-related behaviors to improve physical fitness, physical strength, smoking cessation, and maintenance of an overall healthy lifestyle [85]. In three studies the aim of the intervention was related to psychosocial health. Kloos et al. [81] integrated an online multi-component positive psychology intervention (e.g., positive emotions, discovering and using strength, optimism, self-compassion, and resilience) to increase well-being, and another study investigated the effectiveness of an online mindfulness-based intervention that incorporated elements of mindfulness-based stress reduction programs and mindfulness-based cognitive therapy [89]. Furthermore, Riello et al. [86] developed several types of activities to teach psychological techniques for coping with general stress and to encourage reflection on issues related to work roles and associated emotions.

#### Multimodal intervention approach

The most used approaches combined the organizational and training/educational approaches (*n* = 4). In one of those, the authors introduced a special leadership and management program for middle managers to enable them to deal with the daily realities of nursing services and to create a positive work environment [79]. Furthermore, two STAR (Support, Transform, Achieve, Results) versions comprised supervisor and organizational social support for family and job performance roles to create a healthy psychosocial work environment in RLTC [71, 72]. Pillemer et al. [74] studied the effectiveness of introducing a retention specialist in RLTC. The training included several evidence-based intervention strategies, including peer mentoring, career ladders, communication training, recognition, work-life balance, and enhanced supervision.

Three interventions merged a behavioral and organizational approach [66–69, 83]. The Worksite Heart Health Improvement Project (WHHIP) aimed to reduce the risk of cardiovascular diseases among nursing assistants in RLTC through education about healthy eating, diet, and exercise. The initial version of the WHHIP focused on three components: environment and policy assessment, education of nursing assistants, and ongoing motivation of nursing assistants [68, 69]. The modified WHHIP revised to some extent the content of the initial components and added two new intervention components: technology-based motivation and booster and long-term adherence [66, 67]. The third intervention examined the effectiveness of physical exercise training, stress-management training, and a practical workplace examination (e.g., ergonomics) [83].

### Primary outcomes– demands and resources

Across the included studies mental health and psychosocial parameters were assessed in 15 studies [67, 69–71, 73–76, 78, 82, 83, 86, 88–91]. Of those, three studies examined the effects of health promotion interventions on self-efficacy-related aggressive behavior [70], self-efficacy related to exercise [69], self-efficacy related to healthy eating [69], and self-efficacy related to dementia care [78]. Health, health-related behavior, and well-being were assessed in eight studies [66, 67, 69, 72, 77, 81, 83–85]. Physical parameters were evaluated in four studies [73, 83, 84, 87]. Four studies measured attitudes toward residents’ family/relatives (behavior) [75, 76], the facility [74], residents’ aggressive behavior [70], and residents with dementia [88].

Nine studies investigated outcomes related to the occupation, such as job satisfaction [68, 74, 80, 81, 90, 91], job demands [80], work environment [73, 79], work engagement [81], workplace autonomy [80], workdays loss [73], intention to quit [74, 75], management leadership support [79], work ability [68], sickness absence [83, 84], and turnover rates [74, 79]. Three studies measured the level of RLTC staff knowledge [70, 76, 88].

The classification of primary outcomes into demands and resources in our review is based on the Job Demands-Resources (JD-R) model and a previous review that identified job demands and job resources among nursing staff in hospitals and nursing homes [92]. Among the interventions included in our review, twelve interventions specifically focused on reducing job demands, while only one intervention aimed to enhance the resources of RLTC staff. Furthermore, ten interventions addressed primary outcomes related to both demands and resources, recognizing the importance of considering both aspects in promoting the health and well-being of RLTC staff. All primary outcomes identified in the included studies that are relevant to health and well-being outcomes related to RLTC staff demands and resources are presented in Table 2.

**Table 2:** Identified primary outcomes categorized by job demands and resources of RLTC staff

**Demands**	
• Work pressure	[79]
• Physical comfort	[79]
• Time pressure	[80]
• Mental load	[80]
• Heaviness or stressfulness	[80]
• Effort-Reward-imbalance	[68, 74]
• Demanding contacts with relatives	[75, 76]
• Negative physical and psychological symptoms/demands (health indicators)	[66–69, 71–73, 75, 78, 82–91]
**Resources**	
• Peer cohesion	[79]
• Clarity	[79–81]
• Supervisor support	[79]
• Autonomy	[79, 80]
• Innovation	[79]
• Involvement	[79]
• Task orientation	[79]
• Leadership	[79]
• Work engagement	[81]
• Knowledge	[70, 76, 88]
• Attitudes	[74, 76, 88]
• Career opportunity	[80, 81]
• Contacts with clients and colleagues	[80, 81]
• Performance feedback	[74]
• Self-efficacy	[69, 70, 78]
• Job satisfaction	[68, 74, 80, 81, 90, 91]

### Effectiveness of intervention approaches on health and well-being

#### Training/educational approach

Four out of ten studies (success rate: 40.0%) reported significant results for a training/educational approach [70, 73, 75, 78]. Implementing interventions focusing on work-related competencies in nursing staff showed positive changes in mental health and psychosocial parameters. Specifically, two studies demonstrated improvements in the self-efficacy of nursing staff by implementing a dementia training program with peer support and a training program for managing challenging resident behaviors [70, 78], and O’Brien et al. [73] reported a significant improvement in mental health symptoms. Non-significant results could be observed for health, health-related behavior, and well-being outcomes. Chenoweth et al. [77] did not find a significant improvement in well-being. A group-based acceptance and commitment interventions or cooperative training with relatives appear to be beneficial to enhance staff communication skills, showing positive effects on occupational-related and organizational outcomes, including reduced workday loss and decreased intention to quit work [73, 75]. Moreover, introducing a cooperative communication training program for nursing staff and relatives resulted in a favorable shift in the nursing staff’s attitude toward the behavior of residents’ relatives [75]. Moreover, Irvine et al. [70] observed significant improvements in attitudes, knowledge, and behavioral intentions measured by self-developed items.

#### Behavioral approach

Three out of six studies (success rate: 50.0%) reported significant improvements for a behavioral approach [81, 85, 89]. Mixed results could be observed for health and health-related behavior outcomes. Only one intervention found a significant improvement in body mass index (BMI) using a web- and app-based lifestyle tool [85], while two interventions found no significant improvements [81, 84]. The same result can be observed for occupational-related and organizational outcomes. Only one study found that job satisfaction could be improved through a multicomponent positive psychology intervention [81], while two studies showed no significant improvements [81, 84]. One study identified that an online mindfulness-based intervention positively impacted burnout, both after the intervention and after a three-month follow-up [89]. No significant improvements were observed in physical parameters with two interventions [84, 87].

#### Multimodal intervention approach

Four out of seven studies (success rate: 57.1%) showed significant improvements using a multimodal intervention approach [66–69, 74, 79]. Interventions incorporating both organizational and behavioral components demonstrated enhanced mental health and psychosocial outcomes [66, 69]. Furthermore, significant improvements were observed in various health indicators, including sodium intake, sleep quality, diastolic blood pressure, systolic blood pressure, BMI, physical activity level, cholesterol level, and work ability [66–69]. Two studies employing a combination of training/educational and organizational approaches reported improvements in occupational-related and organizational outcomes [74, 79]. These improvements included enhanced supervisor support, management leadership support, attitudes toward the facility, and reduced turnover rates [74, 79].

## Discussion

This systematic review aimed to provide an overview of the current evidence on health promotion interventions for RLTC staff. This systematic review identified 26 publications presenting 23 individual interventions with a randomized controlled study design during the study period from January 2000 to April 2023. The majority of interventions (*n* = 10) employed a training/behavioral approach, followed by behavioral interventions (*n* = 6). No study exclusively utilized an organizational approach. Additionally, seven studies adopted a multimodal approach that incorporated a combination of at least two single approaches. Out of the included interventions, twelve focused on reducing job demands, while one aimed to enhance the resources of RLTC staff. In ten interventions, both job demands and resources were addressed. Significant improvements were found in nearly half of the included interventions (*n* = 11). The results of this systematic review indicate that health promotion generally has the potential to improve the health and well-being of RLTC staff, but this is highly dependent on the approach and the individual design of the respective intervention.

### Intervention approach

Our research highlights that health promotion for RLTC staff focuses predominantly on the individual level by improving individual work-related competencies in dealing with specific demands of their working environment (e.g., training/educational approach; challenging resident behavior) or trying to change the individual health-related behavior (e.g., behavioral approach; nutrition, physical activity, stress-management). Other systematic reviews have also indicated that the predominant approach to address various health and well-being outcomes, such as physical fitness [47], physical activity [93], body composition [93], and dietary habits [93], is the individual-level approach. In our review, individual-level approaches were most frequently used to address mental health outcomes (*n* = 16), with most interventions using a training/educational approach (*n* = 10). This finding is consistent with the broad landscape of workplace health promotion, as other systematic reviews have examined the effectiveness of mental health interventions in various healthcare professions [51, 94–99]. Moreover, this finding suggests that providing RLTC staff with work-related coping skills empowers them to deal with unpredictable situations in which they may be overwhelmed with demands and may be exposed to constant stressors. Empowerment is a key principle in (workplace) health promotion, as it aims to enhance individuals’ control over their own health and increase their sense of self-determination [100]. Research indicates that a high level of empowerment might have a direct positive impact on various aspects of mental and physical health [101]. A study conducted in the Netherlands demonstrated significant improvements in physical health, behavior change, and mental well-being by targeting menopausal women working in low-paid jobs at a hospital for mental empowerment [102]. Therefore, employee empowerment might have the potential to positively impact employee health and well-being, but it is important to recognize that employees cannot be solely responsible for their own health and well-being in the workplace. In the context of RLTC, working conditions often restrict the nursing staff’s ability to take control over their own health and life. Hence, it is essential to create empowering working conditions encompassing various factors such as information flow, resources, leadership styles, and autonomy to foster a healthy workplace for RLTC staff [103]. Existing research demonstrates that implementing health promotion interventions at an organizational level can be more beneficial for both the individual and the organization by directly addressing the underlying causes of workplace stressors and demands [104, 105]. However, our systematic review identified no interventions exclusively implemented an organizational approach. This result differs from the findings of systematic reviews by Westermann et al. [61] and Romppanen & Häggman-Laitila [106], as the authors identified interventions that applied only an organizational approach (e.g., changing working conditions) to improve symptoms of burnout and well-being among nurses and other healthcare staff. Our review identified that all seven interventions with a multimodal approach contained organizational elements. For instance, three interventions assessed workplace hazards and recommended modifications to the work environment [66–69, 83]. Multimodal intervention approaches can also be found in other nursing setting. The SEEGEN project employed a multimodal intervention approach by integrating tailored behavioral, training/education, and organizational elements for hospital staff [107]. One organizational element of this intervention involved leadership and management training; an element also used in our studies as a part of the multimodal approach. Given the hierarchical structures prevalent in nursing settings, focusing on leadership and management appears crucial.

Beyond the intervention approach, organizational factors are highly relevant in designing and implementing health promotion interventions. Thus, it is crucial to conduct a comprehensive analysis to identify the organizational factors that can be modified through health-promoting measures and those that cannot within the RLTC setting. To achieve this, a shift in awareness and behavior regarding the handling of non-modifiable organizational factors through health promotion measures is required. By conducting a differentiated analysis and implementing tailored interventions that target modifiable organizational factors, the greatest potential for significant improvements in health and well-being among RLTC staff might be realized.

Further, the organizational framework in RLTC settings presents inherent challenges for implementing health promotion interventions. The complexity and rigidity of RLTC create barriers that hinder the successful adoption and effectiveness of such interventions. A review has identified common organizational-level barriers, including lack of time, unsupportive management attitudes towards research, limited resources (e.g., human and financial), a lack of authority to implement practice changes, and a workplace culture resistant to change [108]. The limited availability of human and time resources in LTC settings may contribute to the challenges of implementing new work structures or processes. Additionally, the successful implementation of a health promotion intervention in this context depends on management and staff’s full support and acceptance. These factors, along with the complexity and rigidity of the work conditions and organization, may explain the difficulties in implementing health promotion interventions that aim to modify the organizational framework of RLTC. Future research should address the ambiguity surrounding the utilization and combination of different approaches in implementing health promotion interventions for RLTC staff. Future research should focus on effectively improving modifiable working conditions in RLTC on a larger scale.

### Effectiveness of intervention approaches

Only eleven (out of 23) interventions showed significant improvements. Mental health and psychosocial parameters dominated the endpoints of the included studies, which is consistent with a recent systematic review that examined workplace health promotion interventions for nurses in acute medical care hospitals and LTC facilities in Germany [55]. However, a recently published systematic review, indicates that health promotion in home care primarily emphasizes physical parameters as opposed to our findings [56]. This finding suggests a divergence in the targeted health promotion objectives across healthcare settings.

*Mental health and psychosocial parameters* were significantly improved in five out of 15 interventions (success rate: 33.3%). Those interventions that have proven to be effective included two with a training/educational approach, two interventions with a multimodal approach, and one intervention with a behavioral approach. Two interventions were delivered through online or web-based platforms. For instance, Perez et al. [89] observed significant improvements in burnout and compassion fatigue after a brief adapted online mindfulness-based intervention. The adapted version contributes to the existing literature by suggesting that brief adapted online interventions, considering the organizational barriers (e.g., lack of time) existing in RLTC, might have the potential to improve mental and psychosocial outcomes (e.g., burnout) among the nursing population [109]. This highlights the potential to adapt interventions to the specific context of RLTC, while maintaining the effectiveness.

Furthermore, a review revealed that behavioral interventions effectively reduced burnout in the short-term (six months or less), while a multimodal approach (e.g., a combination of behavioral and organizational approaches) had longer-lasting effects (12 months or more) [110]. One study included in our review, which utilized a behavioral approach, aligns with this finding by showing significant improvements in symptoms of burnout at six weeks and three months after completing the intervention [89]. However, it is worth noting that none of the interventions employing a training/educational approach positively affected burnout. Additionally, none of the interventions utilizing a multimodal approach addressed burnout. Therefore, it is suggested that future studies examine the effectiveness of multimodal approaches in reducing symptoms of burnout among RLTC staff in the short and long term.

*Health, health-related behavior, and well-being outcomes* were significantly improved in four out of eight interventions (success rate: 50.0%). Those interventions that have proven to be effective in our review included two interventions with a multimodal approach, one with a behavioral approach, and one with a training/educational approach. Research has demonstrated that interventions targeting a healthy lifestyle can be beneficial for employees, particularly when they incorporate multimodal intervention components that address both individual behaviors and the work environment [46]. Combining strategies that promote individual behavior change with efforts to create a supportive and health-promoting work environment might enhance the effectiveness and impact of health promotion intervention. The WHHIP program, identified in our review, utilized a multimodal approach that combined education on healthy eating, exercise, and stress-management with structural changes in the work environment and revealed significant improvements in respective outcomes [66–69]. This finding indicates that promoting healthy lifestyles among RLTC staff in the workplace has the potential to improve their health, health-related behaviors, and overall well-being.

*Occupational-related and organizational outcomes* were significantly improved in six out of ten interventions (success rate: 60.0%). Those interventions that have proven to be effective included three interventions with a multimodal approach (one combining behavioral and organizational approaches; two combining training/educational and organizational approaches), two interventions with a training/educational approach, and one intervention with a behavioral approach. This indicates that health promotion interventions targeting RLTC staff have a higher success rate in improving occupational-related and organizational outcomes compared to other outcomes in this review. This finding suggests that the combination of educating RLTC staff to deal with work-related demands and improving the work environment contributed to these positive results. In addition, it is important to recognize that multiple factors influence occupational-related and organizational outcomes. Therefore, multimodal approaches that consider and address these factors may be more effective in achieving positive outcomes. By incorporating multiple strategies and interventions, this approach might effectively address the complexity of these outcomes and enhance the overall effectiveness of health promotion efforts. For example, Pillemer et al. [74] introduced a retention specialist position at a RLTC facility and trained an existing staff member in the interpersonal and management skills required for staff retention. This intervention resulted in significant improvements in intention to quit and turnover rates.

Our systematic review observed that over half of the interventions did not yield significant improvements in health and well-being outcomes among RLTC staff. Research indicates that possessing knowledge about healthy lifestyles does not necessarily translate into healthy lifestyles or behaviors of nurses during their work or leisure time [111]. Behavior change models, such as the Health Action Process Approach (HAPA) model [112] suggest that acquiring better education and knowledge may enhance motivation for engaging in a specific health-related behavior, but external factors such as work conditions may impede the final intention. A recent review identified that most lifestyle health promotion interventions for nurses primarily focus on education [93]. Compared to our systematic review, we observed the same result that most interventions relied primarily on educational approaches delivered in workshops. Formats other than workshops might fit better into the time-limited workday in RLTC. Moreover, integrating concepts of health promotion interventions into the regular workday might have the potential to positively impact both workplace and personal health [113]. One possible approach to incorporating a healthy lifestyle into the workday is through health-promoting activities RLTC staff can engage with residents. This approach might offer several advantages, as it allows RLTC staff to integrate health-promoting activities into their regular work routine, without requiring extra time. Additionally, it benefits both the staff’s own health and contributes to the well-being of the residents they care for. In the context of home care, there is a promising example of home care workers implementing an exercise program for their clients during their visits. The study revealed that home care workers reported significant improvements in their own exercise behavior following the intervention [114]. Hence, future research should explore the impact of an integrated concept to improve health and well-being among RLTC staff.

Another possible reason for limited effectiveness might be that the interventions did not consider the age and gender characteristics of the study participants. In this systematic review, the majority of RLTC staff is female, which is consistent with a publication showing that 83.0% of employees in LTC in Europe are female [4]. None of the included interventions considered gender characteristics according to their reported development process. This finding is consistent with previous research on health promotion interventions among home care workers [56]. Correspondingly, a systematic review revealed that less than 2.0% of health promotion interventions specifically targeted women [115]. If further reasons for limited effectiveness might be associated with the quality of the intervention development process cannot be reliably estimated as intervention studies do not adequately describe the development process of the intervention [116]. Some studies lacked detailed reporting on whether or how the interventions were customized to the specific working conditions, demands, and resources in RLTC. This lack of information about the intervention development hinders replication and makes it difficult to determine if it was evidence-based or followed a logic model [117]. Therefore, it is urgently recommended to consider the specific physiological and sociological characteristics of female RLTC staff and the work conditions’ characteristics when designing interventions for health promotion in RLTC.

### Demands and resources

In our systematic review, we identified twelve interventions that aimed to reduce job demands, one intervention that focused on enhancing job resources, and ten interventions that addressed both job demands and resources. This finding suggests that health promotion interventions for RLTC staff primarily prioritize reducing job demands. However, it is important to recognize that simultaneously addressing both demands and resources is crucial for promoting positive health and work-related outcomes, as emphasized by the Job Demands-Resources model [33]. Therefore, developing a specific core outcome set for health promotion interventions among RLTC staff is recommended, including essential indicators of job demands and resources. This would ensure that future studies and interventions in this context consistently measure and report on these critical aspects, allowing for better comparability and evaluation of the effectiveness of health promotion interventions [118].

### Strength and limitations

The major strength of this study is the provision of the first systematic review of current evidence of health promotion interventions on health and well-being among staff in RLTC. A major limitation of this review is the lack of generalizability, primarily attributed to the wide range of endpoints included and the absence of a core outcome set. In addition, the study group included predominantly female participants and the results might differ for males in this occupational group. Secondly, the focus on only quantitative data meant the exclusion of qualitative data. Rørtveit et al. [119] recommend the additional use of qualitative methods to gain a deeper insight into the complex work environment and conditions of RLTC staff. Third, some studies reported small sample sizes and high attrition rates, which can significantly impact the study results. Fourth, the majority of the included studies lacked methodological quality. Therefore, the results of this review should be interpreted with caution. Fifth, our review may be influenced by publication bias, although it is noteworthy that several studies reported non-significant results. Finally, due to the included studies’ heterogeneous characteristics concerning the interventions’ content and different endpoints measured, no statistical analysis (e.g., meta-analysis) could be performed to estimate the effectiveness of health promotion interventions on a specific outcome for RLTC staff. The results of this systematic literature review were analyzed narratively.

## Conclusions

This systematic review identified 23 different interventions that aimed at improving the health and well-being of staff in RLTC. The current evidence on health promotion interventions for this population is still limited, making it challenging to provide the most effective intervention. However, the review revealed that behavioral, training/educational, and multimodal approaches are potentially beneficial in improving certain outcomes related to staff health and well-being in RLTC. Based on this systematic review, the following recommendations could be made:

- Appropriate design of health promotion interventions is highly relevant in determining their effectiveness and success. Key factors include combining individual-level approaches with organizational approaches, considering empowering working conditions, and tailoring interventions to gender-specific physiological and sociological characteristics of RLTC staff. Developing integrative interventions that can be implemented into RLTC staff’s daily work routines holds the potential to overcome organizational barriers in RLTC, particularly lack of time, which is the most significant organizational barrier to participation in health promotion interventions for RLTC staff.
- A comprehensive description of the development process is essential for the understanding and evaluation of health promotion interventions. Future studies should adequately report the intervention’s development process in terms of evidence and the use of a logic model.
- The use of measurements in the studies varied a lot and this aspect makes it complicated to compare interventions and assess their effectiveness on health and well-being. This underlines a need for further research on well-designed randomized controlled trials of high methodological quality using standardized measurement tools. In addition, a common core outcome set for RLTC staff should be developed that considers the relevant job demands and resources of RLTC staff. Moreover, further research should focus on understanding the interaction between job demand and resources and their impact on health and well-being outcomes among RLTC staff.

### Electronic supplementary material

Below is the link to the electronic supplementary material.

Supplementary Material 1

Supplementary Material 2

## Data Availability

All data generated or analyzed during this study are included in this published article and its supplementary information files.

## Abbreviations

RLTC: residential long-term care
CENTRAL: Cochrane Central Register of Controlled Trials
PRISMA: Preferred Reporting Items for Systematic Reviews and Meta-Analyses
LTC: long-term care
JD-R: Job-Demands-Resources model
PICOS: population, intervention, comparator, outcome, and study design
RoB 2: Risk of Bias assessment tool
BMI: body-mass-index
WHHIP: The worksite heart health improvement project
HAPA: Health Action Process Approach
