# Supplementary material for: Individual and organizational interventions to promote staff health and well-being in residential long-term care: a systematic review of randomized controlled trials over the past 20 years
Source: BMC Nurs. 2024 Mar 22;23:195. doi: 10.1186/s12912-024-01855-7 (PMC10958844; doi:10.1186/s12912-024-01855-7)
Supplement: Supplementary file 1 — Supplementary Material 1 [file 12912_2024_1855_MOESM1_ESM.docx]

**Supplementary Material 1**

**Search Strategy**

Pubmed:

(Intervention [Title/Abstract] OR workshop [Title/Abstract] OR training [Title/Abstract] OR education[mh] [Title/Abstract] OR leadership* OR occupation* OR workplace OR organization* OR "Inservice training" [mh]) **AND** (team* [Title/Abstract] OR nurs* [Title/Abstract] OR *personnel* [Title/Abstract] OR employee* [Title/Abstract] OR staff [Title/Abstract] OR worker* [Title/Abstract] OR *professional* [Title/Abstract]) **AND** ("nursing homes"[mh] OR "nursing home" [Title/Abstract] OR "long-term care*" [Title/Abstract] OR "residential facilities"[mh] OR "residential facility" [Title/Abstract] OR "homes for the aged"[mh] OR "home for the aged" [Title/Abstract] OR "skilled nursing facilities"[mh] OR "skilled nursing facility" [Title/Abstract] OR aged care facilit* [Title/Abstract])

Article type: journal article

Web of Science:

(TS=(Intervention OR workshop OR training OR education OR leadership* OR occupation* OR workplace OR organization* OR "Inservice training")) **AND** (TS=(team* OR nurs* OR *personnel* OR employee* OR staff OR worker* OR professional*)) **AND** (TS=("nursing home*" OR "long-term care*" OR "residential facilit*" OR "home* for the aged" OR "skilled nursing facilit*" OR "aged care facilit*"))

Document types: article

Cochrane Central Register of Controlled Trials

(intervention OR workshop OR training OR education* OR leadership* OR occupation* OR workplace OR organization* OR "Inservice training") AND (team* OR nurs* OR *personnel* OR employee* OR staff OR worker* OR professional* ) AND ("nursing home*" OR "long-term care" OR "residential facilit*" OR "home* for the aged" OR "skilled nursing facilit*" OR "aged care facilit*")

In title/abstract/keyword

APA PsycArticles via EBSCOhost

(Intervention OR workshop OR training OR education OR leadership* OR occupation* OR workplace OR organization* OR "Inservice training" ) AND ( team* OR nurs* OR *personnel* OR employee* OR staff OR worker* OR professional* ) AND ( nursing home* OR "long-term care*" OR "residential facilit*" OR "home* for the aged" OR skilled nursing facilit* OR skilled nursing facilit* OR aged care facilit*)

Scholarly (peer-review) journals Document type: journal article
