# Supplementary material for: Individual and organizational interventions to promote staff health and well-being in residential long-term care: a systematic review of randomized controlled trials over the past 20 years
Source: BMC Nurs. 2024 Mar 22;23:195. doi: 10.1186/s12912-024-01855-7 (PMC10958844; doi:10.1186/s12912-024-01855-7)
Supplement: Supplementary file 2 — Supplementary Material 2 [file 12912_2024_1855_MOESM2_ESM.docx]

**Supplementary Material 2**

**
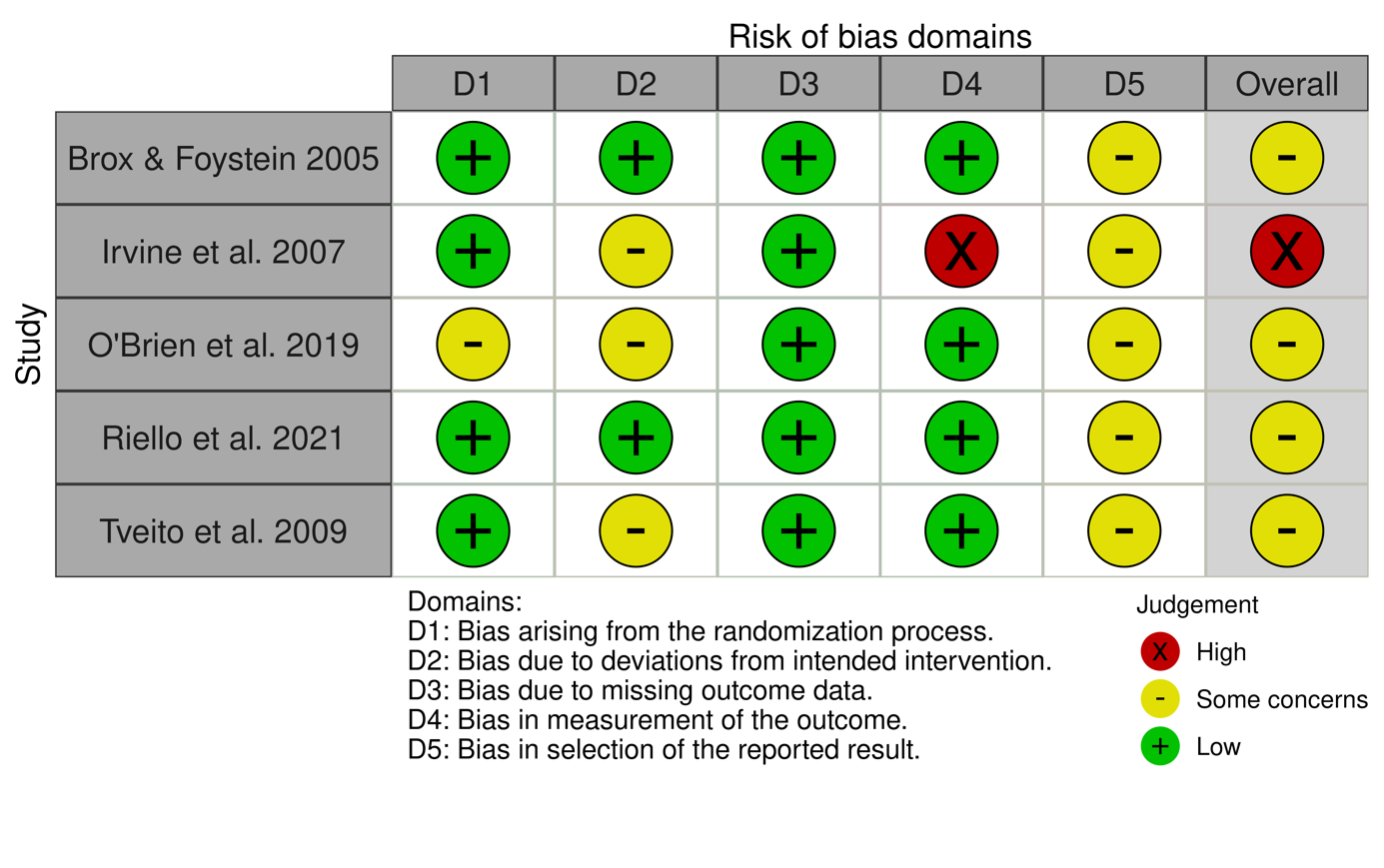
**

S 1 Risk of bias assessment of parallel randomized controlled trials


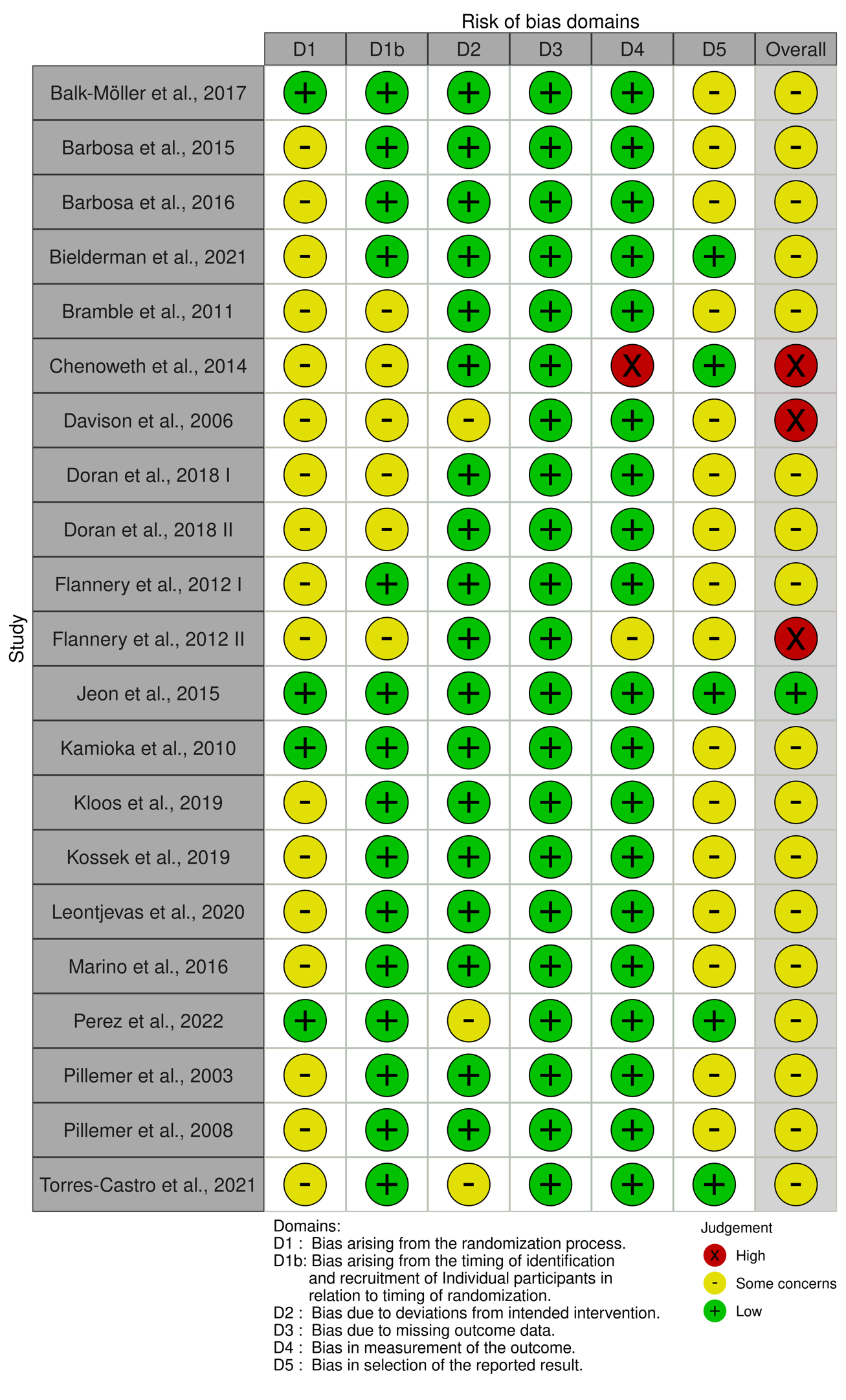


S 2 Risk of bias assessment of cluster randomized controlled trials
